# Rheumatic valvular heart disease combined with woven coronary artery: a case report

**DOI:** 10.1186/s13019-020-01160-9

**Published:** 2020-06-04

**Authors:** Zhengjiang Liu, Yueliang Li

**Affiliations:** Department of Cardiology, Qingyuan People’s Hospital, the Sixth Affiliated Hospital of Guangzhou Medical University, 22 Shuguang Second Road, Qingcheng, Qingyuan, Guangdong 511500 People’s Republic of China

**Keywords:** Woven coronary artery, Rheumatic valvular heart disease, Coronary artery disease

## Abstract

**Background:**

Woven coronary artery (WCA) is an extremely rare congenital anomaly in which a part of epicardial coronary artery is divided into thin channels, that twist along the axis of the coronary arteries and then merge again as the main coronary lumen. This anomaly is regarded as a benign condition because the blood flow is normal. Very few cases of WCA have been reported.

**Case presentation:**

Herein we report a case of a 44-year-old man who was admitted to our hospital due to 20 years of repeated episodes of heart palpitations, 2 years of shortness of breath after activity, and the symptoms were aggravated for 1 month. He had history of inferior myocardial infarction and atrial fibrillation. Color Doppler echocardiography revealed rheumatic heart disease, severe mitral regurgitation, mild-moderate tricuspid regurgitation, moderate pulmonary hypertension. Coronary angiography revealed 60–85% diffuse stenosis in the middle of left anterior descending artery, 60–90% diffuse stenosis in the middle of left circumflex artery, 30–40% diffuse stenosis in the proximal segment of right coronary artery, and WCA anomaly in the middle, and distal segments of right coronary artery.

**Conclusion:**

The patient successfully underwent prosthetic valve replacement and left anterior descending coronary artery bypass grafting, and had a good recovery after surgery. Further studies are needed to fully understand the disease and determine appropriate treatment options.

## Background

Woven coronary artery (WCA), an extremely rare congenital malformation of unclear etiology, was first reported by Sane and Vidaillet in 1988 [1]. The malformation comprises an epicardial coronary artery that is divided into many thin channels that twist along the axis of the coronary artery and then merge to form the main lumen. Either or both left and right coronary arteries can be involved. Blood flow at the distal segment of the WCA is generally normal. One study with a 5-year follow-up showed that no adverse coronary events occurred in patients with a WCA [2]. WCA has been regarded as a benign malformation.

WCA occurs more frequently in men than in women, with the onset concentrated at 39–78 years of age, although WCA was also reported in a 9-month-old male infant who was diagnosed with Kawasaki disease [2]. Several studies have reported that WCAs were associated with myocardial infarction, ischemia, sudden cardiac death, ischemic stroke, and other complications [3–6].

At present, coronary angiography remains the main method for diagnosing WCA. In most cases, WCAs are incidentally detected during coronary angiography performed to determine the condition of coronary blood flow and lesion characteristics. Nevertheless, WCA should be also included in the differential diagnosis of spontaneous coronary artery dissection, intracoronary thrombosis, and chronic total occlusion with bridging collateral vessels.

Rheumatic heart disease (RHD) and atherosclerotic coronary artery disease are common. In patients with RHD, embolism in non-atherosclerotic coronary arteries is the main cause of myocardial infarction. Long-term use of warfarin can reduce the incidence of systemic and coronary embolism in patients with chronic RHD.

We present a rare case of RHD that showed mitral and tricuspid valve regurgitation combined with inferior myocardial infarction due to WCA.

## Case presentation

This study was approved by the ethics committee of Qingyuan People’s Hospital, the Sixth Affiliated Hospital of Guangzhou Medical University. All procedures performed in studies involving human participants were in accordance with the ethical standards of the institutional and/or national research committee and with the 1964 Helsinki declaration and its later amendments or comparable ethical standards. Written informed consent was obtained from the patient included in the study.

A 44-year-old man was admitted to hospital with a 20-year history of repeated episodes of heart palpitations and a 2-year history of shortness of breath after activity. At admission, these symptoms were aggravated and had been present for 1 month. 3 years previously, he had experienced sudden chest pain, with his electrocardiogram indicating inferior myocardial infarction. After undergoing secondary prevention therapy for coronary heart disease (i.e., aspirin, clopidogrel to inhibit platelet aggregation, and atorvastatin to reduce cholesterol levels and improve plaque stability), the patient’s condition improved. 2 years previously, he had atrial fibrillation, for which he was given anticoagulation therapy with warfarin.

**Fig. 1:**
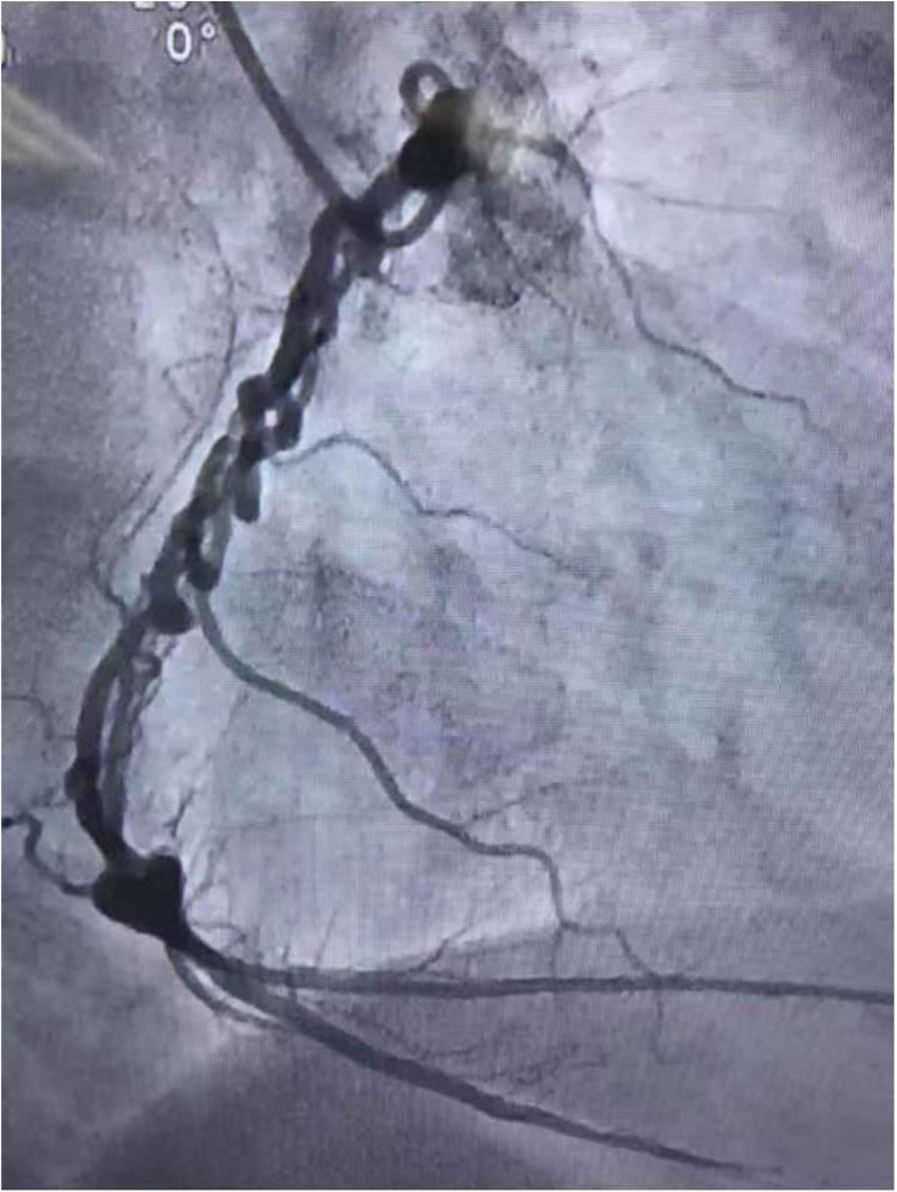
Woven coronary artery anomaly in the right coronary artery

After the present admission, color Doppler echocardiography showed RHD, severe mitral regurgitation, mild-to-moderate tricuspid regurgitation, and moderate pulmonary hypertension. Coronary angiography showed normal right and left coronary ostia and no obvious stenosis in the left main coronary artery. The middle of the left anterior descending artery showed diffuse stenosis (60–85%), with forward flow at grade 3 thrombolysis in myocardial infarction (TIMI). The middle of the left circumflex artery exhibited diffuse stenosis (60–90%), with forward flow at TIMI grade 3. The proximal segment of the right coronary artery was irregular, with 30–40% diffuse stenosis. The WCA anomaly was found in the middle and distal segments of the right coronary artery, with forward flow at TIMI grade 3. The coronary arterial system was right dominant (Fig. 1). The patient was diagnosed with right WCA based on the coronary angiography results.

We replaced the mitral valve with a prosthetic valve and performed single coronary artery bypass grafting on the left anterior descending artery. The left circumflex artery was not grafted because coronary angiography revealed it to be small and unsuitable for bypass grafting. The patient had a good recovery after surgery.

## Discussion

WCA disease is an extremely rare congenital anomaly that may affect either the left or right coronary artery. The right coronary artery is more frequently affected (54.5%), followed by the anterior descending artery (36.4%) and the circumflex artery (9.1%). WCA rarely occurs simultaneously in two (13.6%) or three (4.5%) blood vessels. The extent of the anomaly is limited to a few centimeters. Akyuz et al. [7] reported an average lesion length of 2.2 cm (range 1.0–5.0 cm). Only a few cases of WCA have been reported, and they were primarily in male patients. If WCA is not combined with coronary atherosclerosis, blood flow is mostly normal.

Data on the etiology of WCA in the literature are limited, and the exact pathogenesis of WCA is currently unclear. Uribarri et al. [8] found that the three layers of the arterial wall were intact in WCA patients, and the intimal layer was normal (i.e., not damaged). Therefore, at present, WCA is considered a congenital developmental anomaly of the coronary artery. Soylu et al. [9] showed that spontaneous rupture of the coronary artery can cause pseudotumor-like changes in the artery, with the pseudotumor-like artery gradually forming a true lumen, leading to the separation of the very small coronary artery into several layers and formation of the woven-like structure.

The occurrence of WCA may also be related to angiogenesis and arteriogenesis. Growth factors such as fibroblast growth factor and vascular endothelial growth factor can promote cell division and proliferation. An animal experiment confirmed that these growth factors promote the growth of coronary collateral vessels. It was therefore speculated that intrauterine inflammation and abnormal growth factors may lead to the occurrence of multiple thin channels during coronary artery development [8]. Prior to formation of the coronary artery, there are several blind ends on the aortic wall. When the signal transmission of growth factors is abnormal, these blind ends continue to separate, increase in volume, and form a cavity that develops into a large-diameter arterial blood vessel with blood-conducting functions. The proximal part of the coronary artery is often not affected.

Recanalized thrombus, spontaneous coronary artery dissection, and chronic total occlusion of the coronary artery with bridging collateral vessels are often characterized by a honeycomb- and spiral-like appearance on coronary angiography, similar to characteristic WCA. Hence, WCA may be misdiagnosed when only coronary angiography is performed. Intravascular ultrasonography, optical coherence tomography (OCT), and coronary endoscopy are helpful for determining the structure of the lumen and the walls of the various tunnels, ultimately arriving at a clear diagnosis. OCT is particularly useful for confirming the diagnosis [8].

In the present study, the patient suffered inferior wall myocardial infarction that had produced the chest pain 3 years previously. His condition had improved after appropriate treatment, and there was no recurrent myocardial infarction. It cannot be ruled out, however, that a coronary artery thrombosis had led to the myocardial infarction. After treatment, the coronary artery thrombosis was recanalized, the patient’s myocardial ischemia-related symptoms diminished. The reduced coronary lumen diameter and increased shear stress in the braided segments might accelerate the occurrence and progression of atherosclerosis, causing thrombosis formation and thus myocardial infarction. The alleviation of symptoms after treatment may be associated with recanalization of the coronary artery [2, 6].

## Conclusion

WCA is a rare congenital anomaly. Although it is generally considered benign, interventional cardiologists should keep this anomaly in mind and correctly identify it, especially when coronary artery filling defects are observed, and coronary flow in the distal segments is normal. Angiographic images from multiple projections should be obtained and carefully analyzed. If necessary, OCT or intravascular ultrasonography should be performed to avoid a missed or incorrect diagnosis and to reduce the occurrence of complications associated with unnecessary interventional procedures. In the present study, the patient underwent successful prosthetic valve replacement and left anterior descending coronary artery bypass grafting. However, because of the very low incidence of WCA, few studies have focused on its treatment. Further studies are needed to fully understand the disease and determine appropriate treatment options.

## Data Availability

Not applicable.

## Abbreviations

WCA: Woven coronary artery
RHD: Rheumatic heart disease
CAD: Coronary artery disease
